# Depression and suicide risk prediction models using blood-derived multi-omics data

**DOI:** 10.1038/s41398-019-0595-2

**Published:** 2019-10-17

**Authors:** Youngjune Bhak, Hyoung-oh Jeong, Yun Sung Cho, Sungwon Jeon, Juok Cho, Jeong-An Gim, Yeonsu Jeon, Asta Blazyte, Seung Gu Park, Hak-Min Kim, Eun-Seok Shin, Jong-Woo Paik, Hae-Woo Lee, Wooyoung Kang, Aram Kim, Yumi Kim, Byung Chul Kim, Byung-Joo Ham, Jong Bhak, Semin Lee

**Affiliations:** 1 Korean Genomics Industrialization and Commercialization Center (KOGIC), Ulsan National Institute of Science and Technology (UNIST), Ulsan, 44919 Republic of Korea; 2 Department of Biomedical Engineering, School of Life Sciences, UNIST, Ulsan, 44919 Republic of Korea; 3 Clinomics Inc., Ulsan, 44919 Republic of Korea; 4 Department of Transdisciplinary Studies, Graduate School of Convergence Science and Technology, Seoul National University, Suwon, 16229 Republic of Korea; 5 Division of Cardiology, Department of Internal Medicine, Ulsan Medical Center, Ulsan, Republic of Korea; 6 Department of Neuropsychiatry, College of Medicine, Kyung Hee University, Seoul, Republic of Korea; 7 Department of Psychiatry, Seoul Medical Center, Seoul, Republic of Korea; 8 Department of Biomedical Sciences, Korea University College of Medicine, Seoul, Republic of Korea; 9 Department of Psychiatry, Korea University Anam Hospital, Korea University College of Medicine, Seoul, Republic of Korea; 10 Brain Convergence Research Center, Korea University Anam Hospital, Seoul, Republic of Korea; 11 Personal Genomics Institute, Genome Research Foundation, Cheongju, 28160 Republic of Korea

**Keywords:** Comparative genomics, Predictive markers

## Abstract

More than 300 million people worldwide experience depression; annually, ~800,000 people die by suicide. Unfortunately, conventional interview-based diagnosis is insufficient to accurately predict a psychiatric status. We developed machine learning models to predict depression and suicide risk using blood methylome and transcriptome data from 56 suicide attempters (SAs), 39 patients with major depressive disorder (MDD), and 87 healthy controls. Our random forest classifiers showed accuracies of 92.6% in distinguishing SAs from MDD patients, 87.3% in distinguishing MDD patients from controls, and 86.7% in distinguishing SAs from controls. We also developed regression models for predicting psychiatric scales with *R*^2^ values of 0.961 and 0.943 for Hamilton Rating Scale for Depression–17 and Scale for Suicide Ideation, respectively. Multi-omics data were used to construct psychiatric status prediction models for improved mental health treatment.

## Introduction

Suicide and depression are major health hazards, resulting in the death of one person every 40 s globally^1,2^. They are complex and intertwined phenomena: ~4% of individuals diagnosed with depression commit suicide, and more than half of the persons who attempt suicide meet the criteria of depression^3^. The suicide rate in South Korea (25.8 deaths per 100,000 persons) is among the highest worldwide and is 2.30 times higher than the average of the Organization for Economic Co-operation and Development (OECD) countries (11.2 deaths per 100,000 persons). South Korea has been ranked second among the OECD countries in terms of suicide rates. Notably, the suicide rate for women in South Korea is the highest (14.7 deaths per 100,000 women) among the OECD countries (average 4.86 deaths per 100,000 women)^4^. Hence, predicting depression and suicide risk is a global problem, with exceptional importance in South Korea. Therefore, developing effective models for predicting depression and suicidality may elucidate breakthrough treatments.

**Fig. 1:**
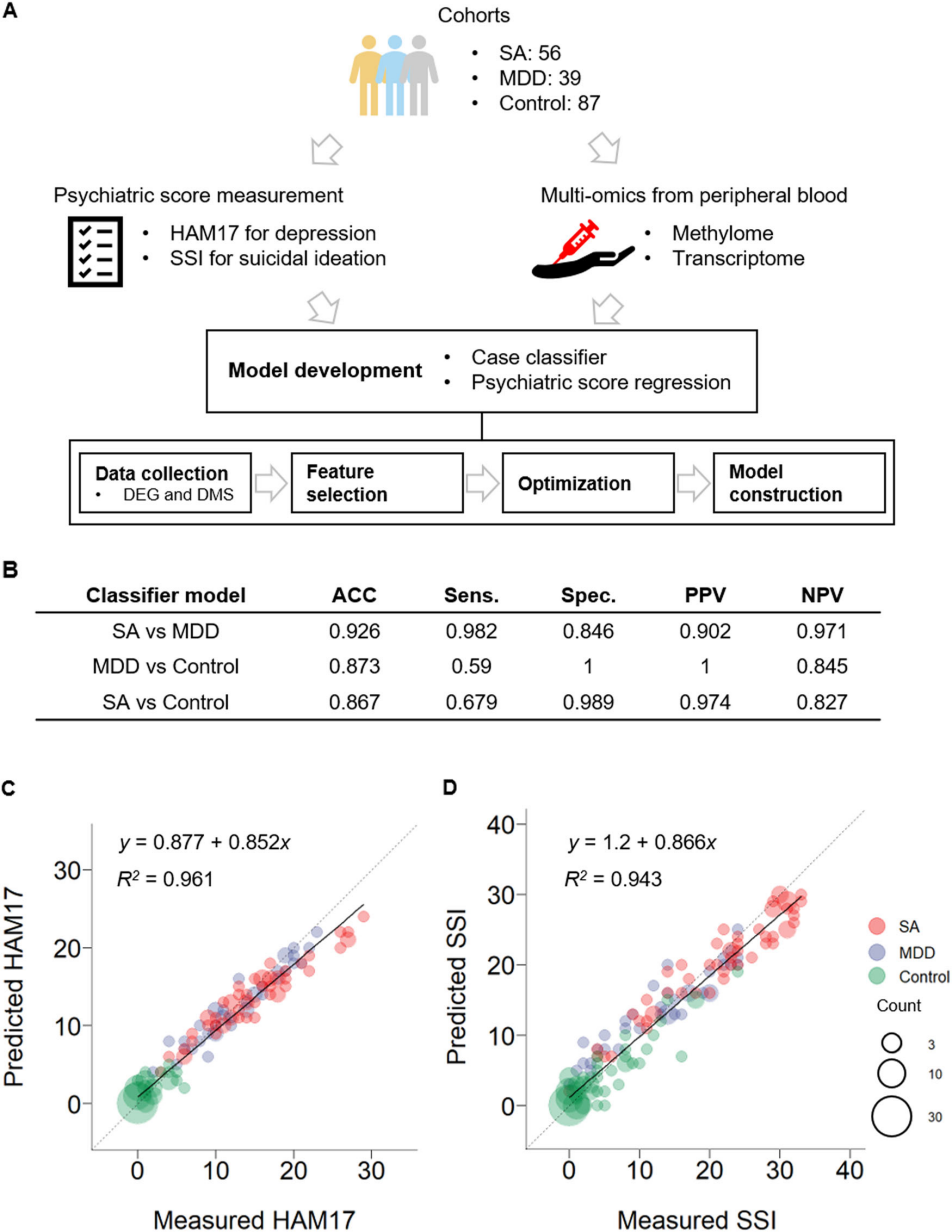
The study workflow and performance of the models. **a** The schema of study workflow, **b** The performances of the case classifier modes, **b**, **c** The performances of the psychiatric score regression models for HAM17 (**c**) and SSI (**d**). SA, Suicide Attempt. MDD, Major depressive disorder. ACC, accuracy. Sens, Sensitivity. Spec, Specificity. PPV, Positive predicted value. NPV, Negative predictive value

The current depression and suicide prediction methods rely on self-reported measures such as questionnaires and interviews, which can be too subjective; and people with depression and suicidal ideation may not be honest about expressing their thoughts^5^. Thus, health records or neural representations have been adopted, with machine learning techniques, to predict the risk of depression and suicide^6,7^. Identifying highly accurate biomarkers would also be an ideal solution that would give an insight to our understanding of depression and suicide. Since the brain is the target organ in psychiatry, brain-based biomarkers have been highly studied^8^. However, an invasive brain biopsy is potentially dangerous, and therefore, biomarkers obtained from the peripheral blood are a practical alternative. Previous studies confirmed meaningful correlations of methylation and expression profiles between the blood and brain^9–11^. Several previous studies identified methylation or gene expression biomarkers for depression and suicide risk from the blood^12–15^. However, none of them combined multi-omics data in a systematic manner to develop models for depression and suicide risk prediction, although applying machine learning to combine different types of multi-omics data may improve prediction accuracy^16–18^. Here, we present machine learning and statistical prediction models for depression and suicide risk prediction using blood-derived multi-omics data (Fig. 1a).

## Results and discussion

### Baseline sample characteristics

We recruited three cohorts (age range: 19–46 years, average: 28.6 ± 8.98 years): (i) 56 suicide attempters (SAs) diagnosed with major depressive disorder; (ii) 39 non-suicide attempters diagnosed with major depressive disorder (MDD); and (iii) 87 healthy individuals (control) through the Korea University Medical Center. Importantly, most of the SA participants (51 of the 56, 91.1%) were recurrent SAs that may also attempt suicide in the future^19^, and 48 out of 56 SA participants had a history of MDD (Tables 1, S1). We collected relevant data from the participants: (i) questionnaires about their history of suicide or depression; (ii) psychiatric scales, including the Hamilton Rating Scale for Depression-17 (HAM17) and the Scale for Suicidal Ideation (SSI); and (iii) peripheral blood samples for methylome and transcriptome sequencing analysis.

**Table 1:** Baseline sample characteristics

Trait	SA	MDD	Control
Number of participants	56 (30.8%)	39 (21.4%)	87 (47.8%)
Average age	31.4 (10.9)	32.1 (11.4)	25.3 (3.5)
Sex, male: female	26:30	21:18	43:44
History of depression	48 (85.7%)	17 (43.6%)	0
History of suicide attempt	51 (91.1%)	0	0
Family history of depression	10 (17.9%)	7 (18.0%)	2 (2.3%)
Family history of suicide attempt	0	1 (2.6%)	0
Antidepressant use	52 (92.9%)	37 (94.9%)	0
HAM17	14.9 (6.0)	13 (5.4)	0.8 (1.4)
SSI	21.6 (8.9)	13.1 (7.3)	3.1 (4.9)

Number (percentage) or mean (s.d) of traits

*SA* suicide attempter, *MDD* major depressive disorder, *HAM17* Hamilton Rating Scale for Depression-17, *SSI* scale for Suicidal Ideation

### Building the psychiatric status classification and regression models

To build the label classification and psychiatric scale regression models, we identified differentially methylated sites (DMSs, *β*-value difference >1% and Benjamini–Hochberg adjusted *P* < 0.05) from Methyl-seq data and differentially expressed genes (DEGs, fold change >1.2 and Benjamini-Hochberg adjusted *P* < 0.05) from whole-transcriptome sequencing data. Next, we performed feature selection to further improve model performance. For the model differentiating SAs from MDD (SA vs. MDD classifier), 7353 DMSs were initially selected, but no DEGs were identified. After the feature selection, 69 DMSs remained (Table S2), and 92.6% accuracy was achieved by leave-one-out cross validation (Fig. 1b). We also selected 12,633 and 10,412 DMSs (16 and 154 DEGs) as input features for the MDD vs. control and SA vs. control classifiers, respectively. After the feature selection, 80 and 95 DMSs (0 and 7 DEGs) remained as input features for the MDD vs. control and SA vs. control classifiers, respectively (Tables S3 and S4). The overall accuracies were 87.3% and 86.7% for the MDD vs. control and SA vs. control classifiers, respectively (Fig. 1b). However, sensitivities were 59% and 67.9% for the MDD vs. control and SA vs. control classifiers, respectively, which were expected. There were no overlapping input features among the classifier models.

**Fig. 2:**
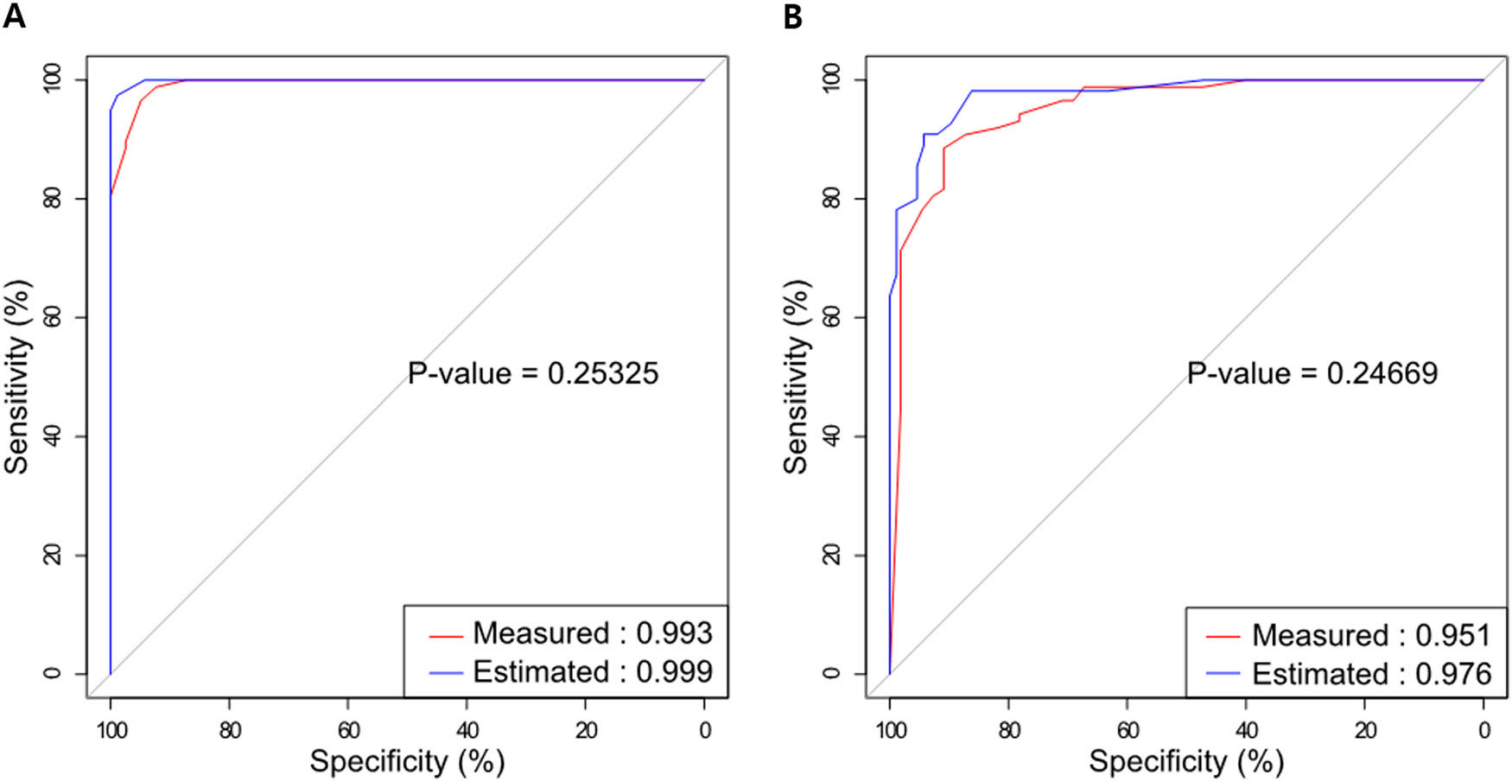
Receiver operating characteristic curves (ROC curve). ROC curves for classifying MDD and Control using the measured and the estimated HAM17 (**a**) and SA and Control using for the measured and the estimated SSI (**b**)

To construct the psychiatric scale regression models, we used the DMSs and DEGs that were significantly correlated (Spearman’s rho > 0.2, *P* < 0.05) with the HAM17 or SSI scores. For the HAM17 regression model, 2150 DMSs and 80 DEGs were selected. For SSI, 1273 DMSs and 82 DEGs were selected. After feature selection, 810 and 467 DMSs (48 and 51 DEGs) remained for HAM17 and SSI regression models, respectively (Tables S5 and S6). There were 139 overlapping markers between the two regression models. *R*^2^ values were 0.961 for HAM17 and 0.943 for SSI (Fig. 1c, d). The area under the receiver operating characteristic curve (AUC)—classifying MDD and control—was 0.993 and 0.999 for the measured and the predicted HAM17, respectively (Fig. 2a). The AUC—classifying SA and control—was 0.951 and 0.976 for the measured and the predicted SSI, respectively (Fig. 2b). The high AUCs from the predicted HAM17 and SSI may compensate for the low sensitivity of the case classifier models for the MDD vs. control and SA vs. control.

### Investigations of the model input features

Since input features were derived from the DEGs and DMSs between groups, investigation of the input feature could give insight into biomarkers significantly associated with depression and suicide attempt. Most of the model input features were methylation markers. This may be due to more methylation markers (DMSs) than gene expression markers (DEGs) from the initial feature selection. Interestingly, the gene expression markers were ranked significantly higher, in terms of feature importance, than the methylation markers only in the regression models (Wilcoxon signed-rank test *P* values for HAM17 regression model: 2.3e-05, SSI regression model: 0.020). Hence, the proportion of marker types in the initial step may not have solely influenced marker types in the final model. This may be due to the relatively more dynamic nature of gene expression levels compared to methylation^20^. Simply, the gene expression markers could more effectively represent emotional state, since the psychiatric assessment was performed together with blood sample collection in this study. However, methylation marker dominance in the classifier models might be due to traumatic experience-related methylation profile changes, as reported previously^21^.

Next, we conducted a functional enrichment test to investigate biological functions and pathways associated with the input features for the models using DAVID (Database for Annotation Visualization and Integrated Discovery)^22^ (Tables 2, S7). No significant enrichment was observed in biological functions or pathways for the SA vs. MDD classifier input features (Benjamini-Hochberg adjusted *P* < 0.05). However, the feature set included the *ARHGAP39* gene (Rho GTPase Activating Protein 39, chr8:145809066, Fig. 3a), a previously reported methylation marker for suicide risk^23^ (Table S2).

**Table 2:** Enrichment analysis result from the models’ makers

Model	Target	Term	Gene number	PCDH gene family	*P*-value	Benjamini *P*
Case classifier	MDD vs. Control	hsa04390:Hippo signaling pathway	6	X	3.70E-04	0.046
Case classifier	SA vs. Control	GO:0007156~homophilic cell adhesion via plasma membrane adhesion molecules	22	O	5.96E-23	3.22E-20
Case classifier	SA vs. Control	GO:0005509~calcium ion binding	25	O	3.86E-12	7.65E-10
Case classifier	SA vs. Control	GO:0005886~plasma membrane	41	O	1.17E-04	0.018
Regression	HAM17	GO:0007156~homophilic cell adhesion via plasma membrane adhesion molecules	40	O	1.01E-20	2.72E-17
Regression	HAM17	GO:0005509~calcium ion binding	69	O	1.75E-11	1.44E-08
Regression	SSI	GO:0007156~homophilic cell adhesion via plasma membrane adhesion molecules	37	O	5.18E-25	1.01E-21
Regression	SSI	GO:0005509~calcium ion binding	51	O	1.11E-11	6.02E-09
Regression	SSI	GO:0007399~nervous system development	25	O	1.52E-07	1.48E-04
Regression	SSI	GO:0045892~negative regulation of transcription, DNA-templated	29	X	3.71E-05	0.024
Regression	SSI	GO:0005886~plasma membrane	134	O	7.94E-05	0.026
Regression	SSI	GO:0003705~transcription factor activity, RNA polymerase II distal enhancer sequence-specific binding	9	X	1.74E-04	0.046

**Fig. 3:**
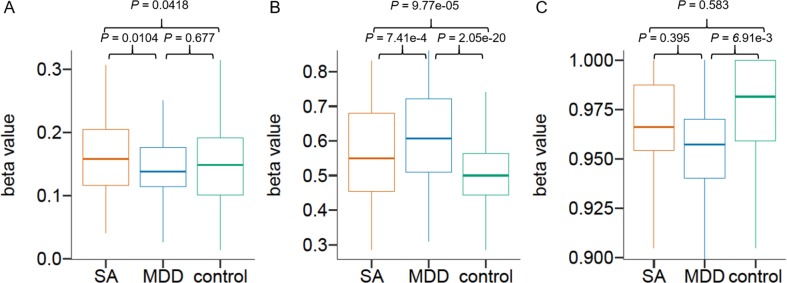
Methylation box plots of the model features. **a** chr8:145809066, *ARHGAP39*. **b** chr2:202900702, *FZD7*. **c** chr1:2010660, *PRKCZ*

We repeatedly observed the protocadherin (PCDH) gene family from enriched biological terms in the feature sets of SA vs. control classifier and HAM17 and SSI regression models (Tables 2, S4–S7). The PCDH gene family is relevant in neuron and synaptic functions, and its methylation can be altered in response to early-life stress^24–27^. A peripheral blood methylation study reported that monozygotic twins that are concordant and discordant for MDDs showed significant intra-pair methylation differences for the *PCDH* genes^28^.

The Hippo signaling pathway was significantly enriched in the MDD vs. control classifier feature set. This pathway includes *PRKCZ* (Protein kinase C, chr2:202900702, Fig. 3b) and *FZD7* (Frizzled Class Receptor 7, chr1:2010660, Fig. 3c), which are known to be related to antidepressant response^29,30^ (Table S3). Although this may be because most (94.9%) of the patients with MDD in this study use antidepressants, it might still suggest antidepressant response as a possible predictor for MDD. This should be validated separately, based on a larger and more diverse cohort.

Here, we present machine learning and statistical models to predict depression and suicide risk, using blood-derived multi-omics data. Our classifier models showed comparable accuracies in predicting the correct labels for patients with MDD, SAs, and healthy controls (Fig. 1b). Psychiatric scales, such as HAM17 and SSI, were also successfully predicted by our regression models (Fig. 1c, d). Although it was marginal, the estimated psychiatric scales classified participants better than the measured scores (Fig. 2a, b). Our models may not guarantee their effectiveness when applied to independent cohorts^31^, but our methodology helps to fill in the gaps in our understanding of the pathogenesis and treatment of psychiatric disorders.

## Methods

### Participant recruitment, diagnostic assessment, and blood sampling

The data in this study presented from three cohorts (i) 56 suicide attempters (SA); (ii) 39 major depressive disorder diagnosed patients (MDD); and (iii) 87 healthy control samples (Tables 1, S1).

A total of 95 depressed patients, with or without suicide attempts were recruited prospectively through the outpatient psychiatric clinic of Korea University Anam Hospital in Seoul, Republic of Korea from April 2015 to August 2017. The groups were then classified to either SA or MDD contingent on the suicide attempt (i.e. 56 suicide attempters and 39 non-suicide attempters). The patients were confirmed with the diagnosis (i.e. major depressive disorder) by the board-certified psychiatrists (Ham BJ, Baek JW and Lee HW) based on the Structured Clinical Interview from the Diagnostic and Statistical Manual of Mental Disorders, Fourth Edition (DSM-IV) Axis I disorders (SCID-I). Basic demographic (e.g. age, sex, education level) and clinical (e.g. antidepressant use, clinical history) information was collected by diagnostic assessments. The current clinical status was measured with psychiatric scales: the Hamilton Rating Scale for Depression-17 (HAM17)^32^ which indicates the severity of depressive symptoms, and the 19-item Beck Scale for Suicide Ideation (SSI)^33^.

There were 10 SAs who were recognized as acute depressive patients with the following criteria: first, those who have current HAM17 score over 14. Second, the duration of current and past suicide attempts was <3 months for those who have the recurrence of suicide attempt (2 out of 10 SAs), or those who attempted suicide for the first time (8 out of 10 SAs).

The healthy controls were recruited for the people between 19 and 65 years of age from the community, in which the advertisements were made. A total of 87 people responded to voluntarily participate in the study. They were assessed through the psychiatric diagnosis in the same way as the patient groups were assessed and determined to have none of psychiatric disorders in past and present.

The diagnostic assessment and blood sampling were made on the same day. The participants’ ID were de-identified after the diagnostic assessment and the blood sampling. In accordance with the Declaration of Helsinki, a total of 182 participants signed informed consents forms about the research goals and procedures. All participants were aware of the right to freely drop out of the study at any stage (no participant dropped out). The study protocol was approved by the Institutional Review Board of Korea University Anam Hospital (IRB No: ED15006). This study was approved by Institutional Review Board at Ulsan National Institute of Science and Technology with UNISTIRB-15-11-C.

### Methyl-seq

Genomic DNA was isolated from blood using the DNeasy Blood & Tissue Kit (Qiagen, Germany) according to the manufacture’s protocol. Extracted DNA was quantified by Quant-iT BR assay kit (Invitrogen). Genomic libraries were prepared using the SureSelect^XT^ Methyl-Seq Target Enrichment System for Illumina Multiplexed Sequencing (Agilent Technologies). Briefly, 2 μg of genomic DNA per sample were randomly sheared via ultra-sonification and DNA fragments between 150 and 200 bp were extracted. Sample DNA then underwent end repair, adapter ligation, hybridization to SureSelect^XT^ Methyl-Seq Capture Library, streptavidin bead enrichment, bisulfite conversion, PCR amplification and were uniquely indexed using a 6-letter sequencing tag following the manufacturer’s protocol. Sample genomic libraries were then pooled and multiplexed in four separate lanes using 100 bp paired-end Illumina NovaSeq6000 S4 sequencing.

### RNA-seq

Total RNA was extracted using PAXgene blood RNA kit from Qiagen (Qiagen, Germany), according to the manufacturer’s recommendations. RNA quality was assessed by running 1 μl on the Bioanalyzer system (Agilent, CA, USA) to ensure RIN and rRNA ratio. We used 100 ng total RNA from all participants to prepare sequencing libraries with by using the TruSeq RNA sample preparation kit (Illumina, CA, USA). Quality of these cDNA libraries was evaluated with the Agilent 2100 BioAnalyzer (Agilent, CA, USA). They were quantified with the KAPA library quantification kit (Kapa Biosystems, MA, USA) according to the manufacturer’s library quantification protocol. Following cluster amplification of denatured templates, sequencing was progressed as paired-end (2 × 100 bp) using Illumina NovaSeq6000 S4 platform.

### Bioinformatic analysis

The sequenced Methyl-seq and RNA-seq read were filtered out when the read’ Q20 base content was lower than 70%, using IlluQCPRLL.pl script of NGSQCToolkit (ver 2.3.3)^34^. The filtered Methyl-seq reads were mapped to the hg19 human genome assembly using Bismark (ver 0.14.5)^35^. Methylation information was acquired using MethylExtract (ver 1.9.1)^36^. The acquired methylation information was further refined as beta value, a proportion of methylated bases at each locus. Only CpG sites with minimum depth ten for equal or more than 75% of samples for both batch and cohort were used. The beta value was adjusted for batch, age, and gender using Combat of SVA package (ver 3.24.4) in R (ver 3.4.0)^37^. The adjusted beta-value was used for further analyses. Differentially methylated site analysis was conducted using methylKit package (ver 1.5.0) in R^38^. All methylation sites were annotated with its positionally related genes (including upstream and downstream 5 kb of gene region). The filtered RNA-seq reads were mapped to the hg19 human genome assembly using Mapsplice (ver 2.1.8)^39^ and gene expression was quantified using RSEM (ver 1.9.1)^40^. The transcripts per kilobase million (TPM) was adjusted for batch, age, and gender using Combat of SVA package (ver 3.24.4) in R (ver 3.4.0)^37^. We identified differentially expressed genes (DEG) using DESeq2^41^.

### Classifier and regression model construction

The three binary classification models (SA vs. MDD, MDD vs. control and, SA vs. control) were constructed using RandomForestClassifier in scikit-learn (ver 0.19.1)^42^. The first step was the feature construction which uses statistical significance of DMS and DEG in each model. DMSs with beta value difference >0.01 and Benjamini-Hochberg adjusted *P* < 0.05; and DEGs with fold change >1.2 and Benjamini–Hochberg adjusted *P* < 0.05 for each comparison (SA vs. MDD, MDD vs. control and, SA vs control) were selected as the feature. Then, the selected features were filtered by feature selection which is the step eliminates the irrelevant features acting as noise to improve the prediction accuracy. For the feature selection, a tree-based feature selection algorithm that calculates feature importance based on the contribution of each feature to model performance during training was used. The features were removed if its feature importance derived from the random forest algorithm during the training was zero. During the training, a number of trees and max features were selected until the out-of-begging (OOB) error rate became stabilized. To verify the model performance, leave-one-out cross validation was used. Two psychiatric scale regression models for HAM17 and SSI were built using LinearRegression in scikit-learn (ver 0.19.1)^42^. The features were selected if the DMSs and the DEGs for each comparison (SA vs MDD, MDD vs Control and, SA vs Control) were significantly correlated with HAM17 or SSI (Spearman correlation rho > 0.2 and *P* < 0.05). We used SelectFromModel in scikit-learn for the feature selection.

### Functional enrichment and pathway analysis

We conducted a functional enrichment test by using DAVID^22^ with default parameters. DEGs and positionally related genes with DMSs from the input feature of the models are used for enrichment test. Only input feature including significant DMSs and DEGs with more than zero feature importance during the model training were selected for functional enrichment test.

## Supplementary information

Supplementary tables's label

Table S1. Baseline sample characteristics (sample by sample)

Table S2. List of selected markers for SA vs MDD classifier model

Table S3. List of selected markers for MDD vs control classifier model

Table S4. List of selected markers for SA vs. control classifier model

Table S5. List of selected markers for HAM17 regression model

Table S6. List of selected markers for SSI regression model

Table S7. Enrichment analysis result of the models' marker

## Data Availability

All sequencing files are available from the National Center for Biotechnology Information (NCBI) database (SRP200298).

## Code availability

Requests for the computer code should be addressed to the author for correspondence.
