# Supplementary material for: Depression and suicide risk prediction models using blood-derived multi-omics data
Source: Transl Psychiatry. 2019 Oct 17;9:262. doi: 10.1038/s41398-019-0595-2 (PMC6797735; doi:10.1038/s41398-019-0595-2)
Supplement: Supplementary file 1 — Supplementary tables's label [file 41398_2019_595_MOESM1_ESM.docx]

**Table S1. Baseline sample characteristics (sample by sample)**

**Table S2. List of selected markers for SA vs MDD classifier model**

Marker info is separated if one marker is related with multiple genes.

**Table S3. List of selected markers for MDD vs control classifier model**

Marker info is separated if one marker is related with multiple genes.

**Table S4. List of selected markers for SA vs. control classifier model**

Marker info is separated if one marker is related with multiple genes.

**Table S5. List of selected markers for HAM17 regression model**

Marker info is separated if one marker is related with multiple genes.

**Table S6. List of selected markers for SSI regression model**

Marker info is separated if one marker is related with multiple genes.

**Table S7. Enrichment analysis result of the models' marker**
